# Antofine Triggers the Resistance Against *Penicillium italicum* in Ponkan Fruit by Driving AsA-GSH Cycle and ROS-Scavenging System

**DOI:** 10.3389/fmicb.2022.874430

**Published:** 2022-04-12

**Authors:** Xuan Peng, Yanan Zhang, Chunpeng Wan, Zengyu Gan, Chuying Chen, Jinyin Chen

**Affiliations:** Jiangxi Key Laboratory for Postharvest Preservation and Non-destruction Testing of Fruits and Vegetables, College of Agriculture, Jiangxi Agricultural University, Nanchang, China

**Keywords:** antofine, Ponkan fruit, induced resistance, blue mold, AsA-GSH cycle, ROS-scavenging system

## Abstract

Postharvest fungal infection can accelerate the quality deterioration of Ponkan fruit and reduce its commodity value. *Penicillium italicum* is the causal pathogen of blue mold in harvested citrus fruits, not only causing huge fungal decay but also leading to quality deterioration. In our preliminary study, antofine (ATF) was found to have a great potential for significant *in vitro* suppression of *P. italicum* growth. However, the regulatory mechanism underpinning ATF-triggered resistance against *P. italicum* in citrus fruit remains unclear. Here, the protective effects of ATF treatment on blue mold development in harvested Ponkan fruit involving the enhancement of ROS-scavenging system were investigated. Results showed that ATF treatment delayed blue mold development and peel firmness loss. Moreover, the increase of electrolyte leakage, O_2_^•–^ production, and malonyldialdehyde accumulation was significantly inhibited by ATF treatment. The ATF-treated Ponkan fruit maintained an elevated antioxidant capacity, as evidenced by inducted the increase in glutathione (GSH) content, delayed the declines of ascorbic acid (AsA) content and GSH/oxidized GSH ratio, and enhanced the activities of superoxide dismutase, catalase, peroxidase, and six key AsA-GSH cycle-related enzymes, along with their encoding gene expressions, thereby maintaining ROS homeostasis and reducing postharvest blue mold in harvested Ponkan fruit. Collectively, the current study revealed a control mechanism based on ATF-triggered resistance and maintenance of a higher redox state by driving AsA-GSH cycle and ROS-scavenging system in *P. italicum*-infected Ponkan fruit.

## Introduction

Ponkan (*Citrus reticulata* Blanco) is an economically important variety of citrus fruit in the world and is very rich in nutrients (e.g., organic acids, vitamin C, dietary fiber, flavonoids, and minerals). It is of full juicy, rounded shape, and pleasant flavor, making it a well-received citrus fruit for consumers in China (Cao et al., 2019; Huang et al., 2021a). However, harvested Ponkan fruit is exceptionally prone to fungal infection by pathogens, such as *Penicillium italicum*, *P. digitatum*, and *Geotrichum citri-aurantii*; among them, *P. italicum* is a causal pathogen of citrus blue mold (BM) during storage, transportation, and market, seriously limiting the storage-life and reducing the commercial value of the harvested fruit (Kanashiro et al., 2020; Duan et al., 2021; Moosa et al., 2021). Currently, controlling citrus BM is generally achieved by postharvest application of synthetic fungicides, which is considered the cheapest and most effective approach to reduce the decay incidence after harvest. However, the long-term use of synthetic fungicides is increasingly causing serious issues limiting their continued use, such as long degradation period, the occurrence of resistant pathogens, environmental pollution, ecological destruction, and increasing public concern about fungicide residues in citrus fruits (Elsherbiny et al., 2021; La Spada et al., 2021; Zhang et al., 2021). In the past decades, resistance induction in postharvest horticultural fruits using plant extracts or their antifungal compounds serving as “generally regarded as safe” (GRAS) additives have shown great potential because they have prominent broad-spectrum antimicrobial activity and will not lead to the emergence of resistant fungal strains and are regarded as a new style alternative to the conventional synthetic fungicides in controlling postharvest fungal decay of citrus fruits (Loi et al., 2020; Oulahal and Degraeve, 2022; Wang et al., 2022).

Generally, the defense and protection mechanisms of fruits can be enhanced by the induction of disease resistance against postharvest fungal infection (Xu et al., 2021). Novel measures have been developed to take no account of synthetic fungicides in the management and control of citrus postharvest fungal disease development, such as edible film (Vera-Guzmán et al., 2019; Huang et al., 2021a), phytohormone (La Spada et al., 2021; Moosa et al., 2021), and biological control (Wang et al., 2022). Among them, the postharvest application of plant-derived antifungal agents (PAAs) to trigger resistance in citrus fruit has been considered as a promising control approach. When invaded by latent fungi, the defense barrier of plant cells was weakened by a disordered reactive oxygen species (ROS) homeostasis due to fungal virulence (Segal and Wilson, 2018; Xu et al., 2019a; Zhang et al., 2020). Nevertheless, over-produced ROS at infection sites can induce oxidative stress, leading to a range of physiological disorders, such as cell permeability breakdown, membrane lipid peroxidation, energy depletion, protein denaturation, and DNA oxidization, thereby resulting in cell death (Zhang et al., 2020; Chen et al., 2022). Postharvest disorder of cellular ROS homeostasis, accompanied by excessive superoxide anion (O_2_^•–^) production and malondialdehyde (MDA) accumulation, will rapidly cause the oxidative damage of cellular membrane as well as cell wall and aggravate the fruit quality deterioration of harvested fruits like Ponkan mandarin (Huang et al., 2021a), orange (Elsherbiny et al., 2021), pummelo (Chen et al., 2022), grape (Wang et al., 2020), pear (Liu et al., 2021), and muskmelon (Xue et al., 2020). To minimize the toxic effects of excessive ROS and reduced oxidative stress, plant cells have evolved an efficient ROS-scavenging system that includes non-enzymatic antioxidants, such as phenolics, flavonoids, ascorbic acid (AsA, also known as vitamin C), glutathione (GSH), and anthocyanins, and enzymatic antioxidants, such as superoxide dismutase (SOD), catalase (CAT), peroxidase (POD), and six key enzymes in AsA-GSH cycle, namely, ascorbate peroxidase (APX), dehydroascorbate reductase (DHAR), mono-DHAR (MDHAR), GSH reductase (GR), GSH peroxidase (GPX), and GSH S-transferase (GST; Hasanuzzaman et al., 2019; Sadeghi et al., 2020; Xue et al., 2020). Therefore, the improvement of the ROS-scavenging system plays a critical role in maintaining ROS homeostasis and conferring host resistance in postharvest fresh crops as a defensive response to fungal infection and abiotic stress.

Increasing evidence has well demonstrated that the inducted resistance by PAAs has become a promising potential strategy in response to the challenge of fungal infections (Chen et al., 2019b; Duan et al., 2021; Li et al., 2021a; Oulahal and Degraeve, 2022). Recently, some PAAs, such as poplar bud extract and its main component, namely, pinocembrin (Yang et al., 2016), citral (Tao et al., 2014), limonin (Li et al., 2021b), salicylic acid, and plant essential oil (Moosa et al., 2021), are effective in the suppression of *P. italicum* growth and in inducing resistance to citrus postharvest blue mold. In addition, Dou et al. (2021) reported that 0.6 g/L epsilon-poly-L-lysine (ε-PL) effectively reduced BM incidence in “Fuji” apple by activating the host-defense responses to *P. expansum* infection. Antofine (ATF; also known as 7-demethoxytylophorine), a natural water-soluble phenanthroindolizidine alkaloid, is known to be mainly isolated from the family of Asclepiadaceae plants and exhibits excellent biological properties, such as anti-angiogenic activity (Oh et al., 2017), cholinesterase inhibitory activity (Othman et al., 2017), antiproliferative and antitumor activities (Yu et al., 2016), and antifungal acidity against Phytopathogen *Fusarium graminearum* (Mogg et al., 2019). In 2019, our group identified six alkaloids (e.g., antofine, 9-dehydroantofine, 9,14-dehydroantofine, 10α-N-oxide-ATF, 10β-N-oxide-ATF, and 14-hydroxy-N-oxide-ATF) from the roots of *Cynanchum atratum* Bunge (Chen et al., 2019a), and ATF was found to have a great potential for significant suppression of *P. italicum* and *P. digitatum* growth (Chen et al., 2019b; Xin et al., 2019). Although ATF exhibits antifungal activity against both the above *Penicillium* spp. under *in vitro* conditions, the resistance enhanced by ATF to control postharvest BM development in citrus fruit has not been investigated yet.

Therefore, our aims in this study were to (1) explore the response of Ponkan fruit toward *P. italicum* infection, (2) elucidate the roles of the induced resistance and ROS metabolism in postharvest BM development in ATF-treated Ponkan fruit infected with *P. italicum*, and (3) evaluate the potential of ATF treatment as an effective strategy for preventing or controlling citrus BM and enhancing the storability of harvested Ponkan fruit.

## Materials and Methods

### Fungal Pathogen and Agentia

The fungal pathogen of *P. italicum* was isolated from an infected citrus fruit with typical BM symptoms (Chen et al., 2019b) and maintained on potato dextrose agar (PDA) medium at 25 ± 1°C. A spore suspension of *P. italicum* was prepared with sterile distilled water and adjusted to a final concentration of 5.4 × 10^5^ spores/ml with the aid of an automatic cell counter.

Antofine used in this study was extracted and isolated from the rhizome of *C. atratum* Bunge in our laboratory (Jiangxi Province, China) (Chen et al., 2019a) and was dissolved in sterile distilled water for subsequent experiments.

### Fruit Treatment

Mature fruits of Ponkan were harvested at a commercial orchard from Jing’an city, Jiangxi Province, China (latitude 28.86°N and longitude 115.36°E). After removing field heat for 48 h, fruits with uniform size (140–160 g per fruit) and ripeness [citrus color index (CCI): 5.12–5.48 and total soluble solids (TSS)/titratable acid (TA): 7.22–7.67] were selected for experiments. The results from our preliminary experiment showed the minimum fungicidal concentration (MFC) value of ATF against *P. italicum* was 6.25 mg/L (Chen et al., 2019b). The *in vivo* inhibitory effects of ATF at different concentrations [0 × (control), 1.25 ×, 2.5 ×, 5 ×, 10 × MFC] for controlling postharvest BM development on Ponkan fruit was disposed by adopting our previous method described by Chen et al. (2022). For each Ponkan fruit, one wound (diameter of 3 mm and depth of 3 mm) was made using a sterilized punch. Initially, a 15 μl of 62.5 mg/L ATF solution or distilled water (control) was added to each wound. After air-drying for 1 h, an equal volume (15 μl) of fungal spore suspension was reinjected. All *P. italicum*-infected fruits were put in plastic boxes and then stored at 25 ± 1°C with relative humidity over 95%. The *in vivo* experiment was performed twice, with three replicates of 10 fruits for each group.

### Sample Collection

Healthy peel tissues of 15–20 mm were collected surrounding the water-soaked lesion with a disinfected scalpel. 45 fruits per treatment, 15 fruits from each replicate in the control, and 62.5 mg/L ATF-treated Ponkan fruit were sampled at 0, 12, 24, 48, 72, and 96 h. All samples were rapidly frozen in liquid nitrogen, ground into powder, and stored at –80°C for the next physical-biochemical analysis.

### Determination of Disease Development, Fruit Firmness, Electrolyte Leakage, O_2_^•–^ Production Rate, and Membrane Lipid Peroxidation in Peel Tissues of *Penicillium italicum-*Infected Ponkan Fruit

The diameter of water-soaked lesion on the *P. italicum*-infected Ponkan fruit was used as one of the direct indexes of blue mold development and recorded in millimeter (mm) in the initial stage of infection progress (Chen et al., 2022).

Peel firmness (N) of 10 fruits from two *P. italicum*-infected groups was determined by using a TAXT Plus texture tester (SMS, Godalming, United Kingdom) fitted with a 2 mm diameter probe, with the results expressed as Newton (N). For the assessment of membrane stability, electrolyte leakage in Ponkan peel was assayed by the method elaborated by Chen et al. (2022) with the aid of a portable conductivity meter (DDS-307A, Leici Inc., Shanghai, China). The hydroxylamine method elaborated by Sadeghi et al. (2020) was applied to determine superoxide anion (O_2_^•–^) production rate, and the value was expressed as μmol^–1^ min g^–1^. As the end product is lipid peroxidation, MDA content was determined by referring to the modified thiobarbituric acid (TBA) method as previously proposed (Gong et al., 2021).

### Assay of Antioxidant Capacity

The hydroxyl radical (•OH) scavenging activity was applied to assess the antioxidant capacity of the control and ATF-treated peel tissues and assayed in line with the method described (Chen et al., 2022). The unit of percentage (%) represented the •OH scavenging activity.

### Measurement of Intermediate Metabolite Content in Ascorbic Acid-Glutathione Cycle and Antioxidant Enzyme Activities

After a 96 h of *P. italicum*-infected period, two ascorbates of AsA and oxidized AsA (DHA) reduced glutathione (GSH) and its oxidized GSH (GSSG) in both control and ATF-treated peel tissues that were assayed in line with the method described (Sadeghi et al., 2020). The contents of AsA, DHA, GSH, and GSSG were expressed as mg kg^–1^ frozen weight (FW).

Meanwhile, to determine all antioxidant enzyme activities, 3.0 g of peel tissues from the control and ATF-treated Ponkan fruit was homogenized with 10 ml of 50 mM ice-cold phosphate buffer [pH 7.5, 1 mM EDTA, and 2% polyvinylpyrrolidone (PVP)], and then centrifuged for 30 min at 4°C with the centrifugal force of 12,000 × *g*. The supernatant was collected cautiously and used for assaying the activities of SOD (EC 1.15.1.1), CAT (EC 1.11.1.6), POD (EC 1.15.1.1), APX (EC 1.11.1.11), GR (EC 1.8.1.7), MDHAR (EC 1.6.5.4), DHAR (EC 1.8.5.1), GPX (EC 1.11.1.9), and GST (EC 2.5.1.18).

Superoxide dismutase activity was assayed using a commercial SOD kit (No. A001-1-2, Nanjing Jiancheng Bioengineering Institute, China) for recording the absorbance at 550 nm, and it was expressed as U g^–1^ FW. The activities of CAT, POD, APX, GR, and GPX were measured by the reported method of Chen et al. (2022), with the absorbance recorded at 240, 420, 290, 340, and 470 nm, respectively. In addition, MDHAR activity was measured by adding 1.5 mL of enzyme extract to initiate a reaction solution containing 2.0 ml of 50 mM phosphate buffer, 0.2 ml of 0.2 mM NADPH, and 0.2 ml of 10 mM AsA (Zhu et al., 2022). Similarly, DHAR activity was assessed using a reaction solution (4.0 ml) containing 2.6 ml of 50 mM phosphate buffer, 0.3 ml of enzyme extract, 0.4 ml of 2 mM GSH, and 0.3 ml of 10 mM EDTA, with 0.4 ml of 500 mM DHA for reaction. One unit (U) of MDHAR and DHAR activities was expressed as 0.01ΔOD_340_ g^–1^ and 0.01ΔOD_265_, respectively. GST activity in peel tissue was estimated by detecting the increase in the absorbance at 340 nm of a reaction mixture (1.0 ml) containing 0.5 ml of 100 mM Tris–HCl buffer [pH 6.5, 1.5 mM GSH, and 1.0 mM 2,4-dinitrochlorobenzene (DNCB)] and 0.5 ml of enzyme extract (Mostofa et al., 2014). GST activity was defined as GSH reduced per minute and expressed as U g^–1^ FW.

### Analysis of Related Gene Expression by Quantitative Real-Time PCR

Total RNA was extracted from 0.5 g frozen peel tissue from the control and ATF-treated Ponkan fruit following the procedures described by Ma et al. (2014). The quality and quantification of RNA were determined using 1.0% agarose gel electrophoresis and a NanoDrop 2000 spectrophotometer, respectively. The synthesis of first-strand cDNA was performed using a Hifair II first-strand cDNA synthesis kit (Yeasen, Shanghai, China). The gene expression patterns of *MnSOD1* (Ponkan7g_002820), *CAT1* (Ponkan2g_014350), *POD1* (Ponkan4g_000980), *APX1* (Ponkan2g_006070), *GR2* (Ponkan5g_040100), *MDHAR5* (Ponkan5g_022520), *DHAR3* (Ponkan7g_004490), *GST3* (Ponkan5g_037340), and *GPX2* (Ponkan5g_023250) in Ponkan fruit were analyzed by qRT-PCR and designed using Primer Express 5.0. The citrus β*-actin* (Ponkan1g_003790) gene was used as the internal control gene for each gene amplification. All qRT-PCR was performed triplicates in a 10-μl mixture containing 1.0 μl of diluted cDNA, 0.3 μl of each gene-specific primer (1 μM, Supplementary Table 1), 5 μl of TB Green Premix Ex Taq and 3.4 μl of nuclease-free water. The PCR protocol was as follows: an initial denaturation at 95°C for 1 min, followed by 40 cycles at 95°C for 15 s, and a primer extension at 63°C for 25 s. The relative expression of each targeted gene was calculated by the 2^–ΔΔCt^ method with three biological replicates.

### Statistical Analysis

Each assay was performed in triplicate to ensure the validity of the experimental data and to reduce the variance. The differences of the mean between individual treatments were analyzed by one-way ANOVA, followed by paired-samples *T*’s test at the level of *P* < 0.05 or *P* < 0.01.

## Results

### Antofine Delayed Blue Mold Development in Ponkan Fruit to *Penicillium italicum* Infection

**FIGURE 1:**
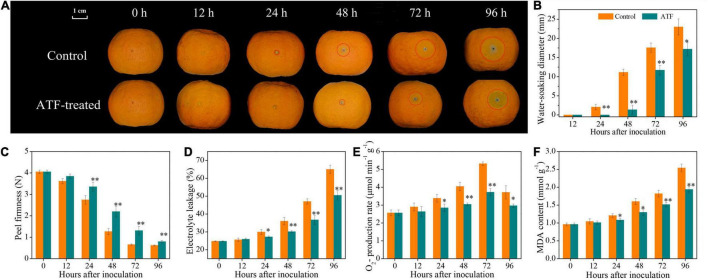
The mitigating effects of ATF treatment on blue mold development **(A)**, water-soaking diameter **(B)**, firmness **(C)**, electrolyte leakage **(D)**, O_2_•^–^ production rate **(E)**, and MDA content **(F)** in harvested Ponkan fruit response to *P. italicum* infection. The asterisk of * (*P* < 0.05) or ** (*P* < 0.01) within the same time point denotes a significant difference among two *P. italicum*-infected Ponkan fruits.

As shown in Figure 1A, *P. italicum* infection accelerated blue mold development in postharvest Ponkan fruit. Early evidence of water-soaking appeared in the control wound during the initial stage of *P. italicum* inoculation. Moreover, a remarkable increase in the water-soaking diameter was found as lesion occurred in the control wound after 24 h of *P. italicum* inoculation, whereas this occurrence was considerably delayed or decreased following the ATF treatment (Figure 1B).

### Firmness Loss and Oxidative Damage *in Penicillium italicum-*Infected Ponkan Fruit Were Prominently Reduced by the Antofine Treatment

Peel firmness decreased in two *P. italicum*-infected Ponkan fruits, but no significant difference was observed between the control and ATF-treated groups during the first 24 h of *P. italicum* inoculation (Figure 1C). At 48 h, the peel of *P. italicum*-infected fruit treated with ATF still retained higher peel firmness than the control Ponkan fruit. Compared to the control Ponkan fruit, the ATF-treated fruit showed a prominent suppression in firmness loss, indicating that exogenous ATF treatment was found to have a great potential for the maintenance of the integrity of cell wall mechanics.

The electrolyte leakage in two *P. italicum*-infected Ponkan fruit exhibited an uptrend all over the inoculation duration (Figure 1D). The electrolyte leakage in the control Ponkan fruit increased from an initial value of 24.8% to a maximum value of 65.0% at the end of *P. italicum* inoculation (96 h). ATF treatment significantly inhibited the ascent of electrolyte leakage, in which the electrolyte leakage at 96 h was 22.4% lower than that of the control Ponkan fruit.

As shown in Figure 1E, the rate of O_2_^•–^ production in the control Ponkan fruit increased steadily within the first 72 h, followed by a sharp decrease at 96 h of *P. italicum* inoculation. In contrast, the ATF-treated Ponkan fruit exhibited a slight variation during the entire period of inoculation. It was obvious that the overall O_2_^•–^ production rate in the ATF-treated Ponkan fruit was significantly lower (*P* < 0.05) than those of the control.

An increasing accumulation of MDA content was recorded in two *P. italicum*-infected Ponkan fruits; however, the increase was significantly retarded by ATF treatment (Figure 1F). A lower (*P* < 0.05) accumulation of MDA content in the ATF-treated group could be attributed to the slowing down in lipid peroxidation caused by *P. italicum* infection.

### Effects of Antofine Treatment on Ascorbic Acid-Glutathione Cycle Components in *Penicillium italicum-*Infected Ponkan Fruit

**FIGURE 2:**
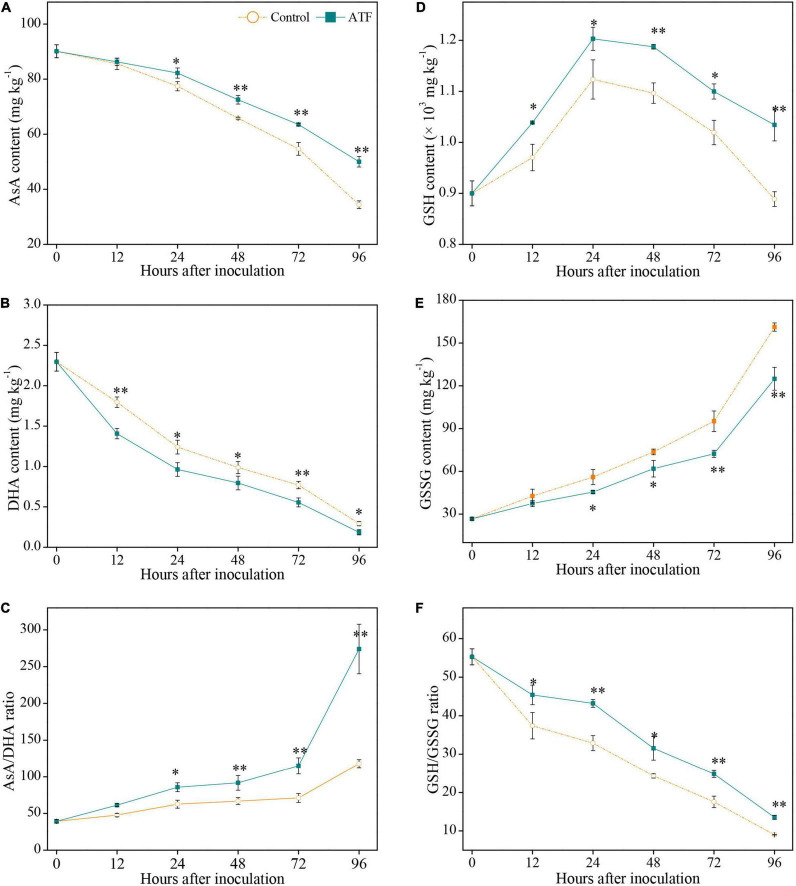
Variation in AsA content **(A)**, DHA content **(B)**, AsA/DHA ratio **(C)**, GSH content **(D)**, GSSG content **(E)**, and GSH/GSSG ratio **(F)** in the peel of *P. italicum*-infected Ponkan fruit treated with or without ATF during the postinoculation period. The asterisk of * (*P* < 0.05) or ** (*P* < 0.01) within the same time point denotes a significant difference among two *P. italicum*-infected Ponkan fruits.

The AsA content in two groups declined gradually with the inoculation duration of *P. italicum*, and the ATF treatment had a prominent inhibition for the degradation of AsA in *P. italicum*-infected Ponkan fruit (Figure 2A). Similar to the overall variation of AsA content, DHA content in both groups exhibited a downturn trend all over the process of *P. italicum* inoculation (Figure 2B), with a more pronounced decline of DHA content in the ATF-treated Ponkan fruit. The ratio of AsA/DHA is related to the redox state of Pokan peel response to *P. italicum* infection. The AsA/DHA ratio in the ATF-treated Ponkan fruit was higher during the inoculation from 0 to 96 h compared with that in the control Ponkan fruit (Figure 2C). At 96 h of *P. italicum* inoculation, the AsA/DHA ratio in the ATF-treated group was about 2.0 times higher than the control fruit. A higher (*P* < 0.05) level of AsA/DHA ratio in the *P. italicum*-infected Ponkan fruit treated with ATF may be related to the increased redox state and the enhanced resistance against *P. italicum* infection in Ponkan fruit.

The GSH content in two *P. italicum*-infected Ponkan fruits showed an upward trend within the first 24 h of *P. italicum* inoculation and then declined (Figure 2D). It is worth mentioning that ATF treatment resulted in activated GSH content, being an overall 1.2 times higher than that in the control fruit throughout the process of *P. italicum* inoculation. The GSSG content in the control Ponkan fruit increased with the progress of *P. italicum* inoculation, being 6.1 times higher than the initial value of 26.6 mg kg^–1^. Compared to the control group, ATF treatment remarkably restrained the process of GSSG accumulation (Figure 2E). Exposure to ATF treatment resulted in 22.4% inhibition of GSSG accumulation for the control Ponkan fruit at the end (96 h) of *P. italicum* inoculation. A continuous decline in GSH/GSSG ratio was observed in the control Ponkan fruit during the whole *P. italicum* infection; however, Ponkan fruit exposed to ATF treatment possessed a higher GSH/GSSG ratio than those in the control (Figure 2F).

### Effects of Antofine Treatment on Antioxidant Capacity in *Penicillium italicum-*Infected Ponkan Fruit

**FIGURE 3:**
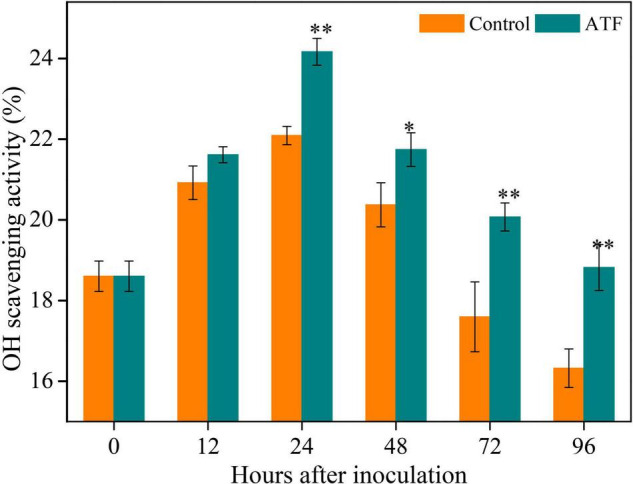
Variation in antioxidant capacity in the peel of *P. italicum*-infected Ponkan fruit treated with or without ATF during the postinoculation period. The asterisk of * (*P* < 0.05) or ** (*P* < 0.01) within the same time point denotes a significant difference among two *P. italicum*-infected Ponkan fruits.

The •OH scavenging activity is the important indicator of total non-enzymatic antioxidant capacity. The •OH scavenging activity in Ponkan peel from both groups peaked at 24 h, followed by a gradual decrease during the rest of *P. italicum* inoculation (Figure 3). Compared to the control group, ATF treatment first increased the •OH scavenging activity and then remarkably delayed the following decline.

### Effects of Antofine Treatment on the Activities and Gene Expressions of Superoxide Dismutase, Catalase, and Peroxidase in *Penicillium italicum-*Infected Ponkan Fruit

**FIGURE 4:**
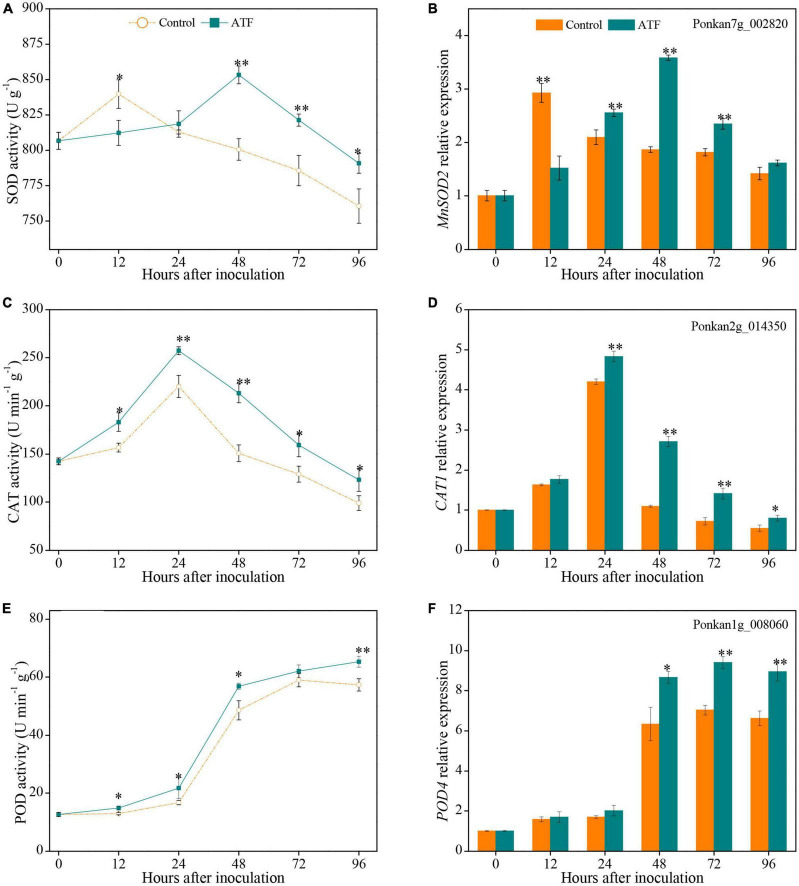
The activities and expression levels of SOD **(A,B)**, CAT **(C,D)**, and POD **(E,F)** in the peel of *P. italicum*-infected Ponkan fruit treated with or without ATF during the postinoculation period. Gene expression of the peel sample from 0 day was defined as 1. The asterisk of * (*P* < 0.05) or ** (*P* < 0.01) within the same time point denotes a significant difference among two *P. italicum*-infected Ponkan fruits.

Two peaks of SOD activity in the control and ATF-treated Ponkan fruit were assayed at 12 h and 48 h, respectively (Figure 4A). ATF treatment delayed the peak for 36 h and increased SOD activity than that in the control Ponkan fruit during the last 72 h of *P. italicum* inoculation. Exposure to ATF treatment significantly increased the expression level of *MnSOD2* at 24–72 h compared to the control group (Figure 4B).

Catalase activity in both *P. italicum*-infected fruits showed a similar trend, reaching the peak at 24 h (Figure 4C). ATF treatment stimulated the increase of CAT activity and delayed its decline, which resulted in a higher overall CAT activity by 23.9% compared to the control Ponkan fruit from 12 h to 96 h. Interestingly, *CAT1* expression was also notably increased by ATF treatment after 12 days of *P. italicum* inoculation (Figure 4D). The peak of *CAT1* expression was determined at 24 h as 17.8% higher in *P. italicum*-infected fruit treated with ATF than that in the control.

Peroxidase activity in both sets increased with the progress of *P. italicum* infection. ATF treatment enhanced the increase of POD activity during the whole inoculation period, with 14.0% higher POD activity than that of the control Ponkan fruit at 96 h (Figure 4E). Exposure to ATF treatment remarkably increased *POD4* expression during the last 48 h of *P. italicum* inoculation (Figure 4F). The *POD4* expressions in the ATF-treated Ponkan fruit at 48, 72, and 96 h were 36.6, 33.9, and 35.0%, respectively, higher than those in the control group.

### Effects of Antofine Treatment on Ascorbic Acid-Glutathione Cycle-Related Enzyme Activities and Gene Expressions in *Penicillium italicum*-Infected Ponkan Fruit

**FIGURE 5:**
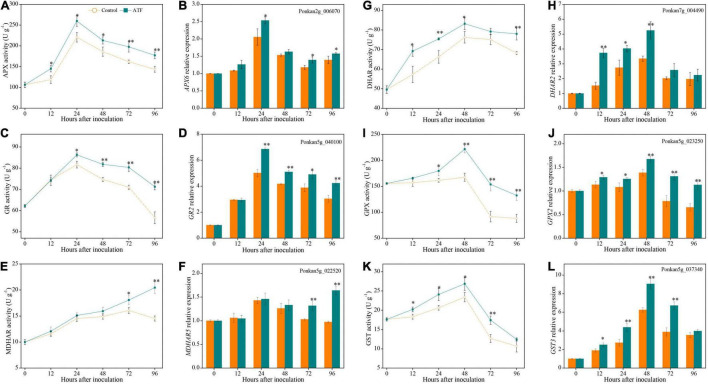
The activities and expression levels of APX **(A,B)**, GR **(C,D)**, MDHAR **(E,F)**, DHAR **(G,H)**, GPX **(I,J)**, and GST **(K,L)** in the peel of *P. italicum*-infected Ponkan fruit treated with or without ATF during the postinoculation period. Gene expression of the peel sample from 0 day was defined as 1. The asterisk of * (*P* < 0.05) or ** (*P* < 0.01) within the same time point denotes a significant difference among two *P. italicum*-infected Ponkan fruits.

The activities of APX and GR peaked at 24 h after *P. italicum* inoculation in Ponkan fruit, for both the control and ATF-treated sets (Figures 5A,C). Additionally, the noteworthy high levels of APX and GR activities were found in the ATF-treated Ponkan fruit compared to the control in the late stage (from 24 to 96 h). Simultaneously, the expression levels of *APX6* and *GR2* followed a trend similar to the overall activities of APX and GR, reaching their expression peak at 24 h (Figures 5B,D).

Mono-DHAR activity exhibited an overall increasing trend during the process of *P. italicum* inoculation (Figure 5E). ATF treatment elevated the increase of MDHAR activity in *P. italicum*-infected Ponkan fruit, being 40.7% higher than that of the control at the end of *P. italicum* inoculation. However, during the first 48 h of the inoculation period, there was no evident difference in the expression level of *MDHAR5* gene between the control and ATF-treated groups (Figure 5F).

Dehydroascorbate reductase activity increased sharply in the early stage of *P. italicum* inoculation and reached its peak at 48 h, followed by a slight decrease (Figure 5G). ATF treatment promoted the increase and suppressed the decline of DHAR activity, which resulted in a noteworthy higher level of DHAR activity (by 14.2% on average) compared with the control Ponkan fruit from 12 to 96 h. Moreover, ATF treatment greatly enhanced the upregulated expression level of *DHAR2* gene during the first 48 h of the inoculation period (Figure 5H).

Similar to the overall changes of DHAR activity, both GPX and GST activities in two *P. italicum*-infected Ponkan peel increased during the first 48 h of *P. italicum* inoculation and dropped sharply thereafter (Figures 5I,L). However, GPX activity at 24–96 h and GST activity at 12–72 h were significantly higher in the ATF-treated Ponkan fruit than those in the control. ATF treatment immediately triggered a remarkable increase in the expression levels of *GPX2* and *GST3* gene and reached the two expression peaks at 48 h, which were 1.45 and 1.21 times higher than those in the control Ponkan fruit, respectively (Figures 5J,K).

## Discussion

Postharvest production of citrus fruits has been plagued by the fungal infection caused by various pathogens, such as *Penicillium* spp. (blue and green mold), *Geotrichum citri-aurantii* (sour rot), *Colletotrichum acutatum* (anthracnose), and *Diaporthe citri* (*Phomopsis* stem-end rot) (Kanashiro et al., 2020; Duan et al., 2021; Elsherbiny et al., 2021; Muñoz-Guerrero et al., 2021). As promising alternatives to conventional synthetic fungicides, PAAs have received increasing attention due to their great potential for controlling postharvest fungal decay and maintaining the nutritional quality of citrus fruits. Our earlier studies demonstrated that ATF could control *Penicillium* spp. decay in citrus fruit, possibly mainly by the direct inhibition of *Penicillium* spp. growth (Chen et al., 2019b; Xin et al., 2019). However, it is largely unknown how ATF indirectly induces resistance in citrus fruit response to *P. italicum* infection. In this work, we first carried out an *in vivo* test to evaluate the control efficacy of ATF at 0 ×, 1.25 ×, 2.5 ×, 5 × and 10 × MFC as natural elicitors to trigger host resistance in *P. italicum*-infected Ponkan fruit. The *in vivo* results showed that ATF treatment significantly delayed blue mold development and water-soaking process caused by *P. italicum* infection in Ponkan fruit (Figure 1A and Supplementary Figure 1), and ATF treatment at 62.5 mg/L (10 × MFC) was the most effective. However, as far as we know, the applied dosage of imazalil for controlling citrus blue mold (*P. digitatum*) decay was recommended not to exceed 2 g/L (Erasmus et al., 2015). Therefore, it is well demonstrated that ATF could be a promising PA for the effective management of postharvest *Penicillium* spp. diseases in citrus fruit.

Since the cell membrane and its wall in plants are considered to be the unique physical barriers in plant-fungal pathogen interactions, the integrity of the cell membrane and its wall structure is vitally important for conferring host resistance in horticultural crops against fungal infection (Segal and Wilson, 2018; Villa-Rivera et al., 2021). Therefore, the imbalance of cell integrity may rapidly aggravate the development of disease decay in fungal-infected fruits. The huge loss of peel firmness is the ubiquitous evidence of lesion in many horticultural fruits response to biotic stress or fungal infection (Ma et al., 2019; Chen et al., 2022), which is largely associated with the peroxidation of membrane lipid and the polysaccharide components disassembly or degradation from its cell wall (Sadeghi et al., 2020; Xue et al., 2020). Electrolyte leakage, O_2_^•–^ production rate, and MDA (the final product in membrane peroxidation) content have been widely considered as key important indicators for assaying the degree of cell oxidative damage in fruits in response to postharvest fungal infection (Marschall and Tudzynski, 2016; Abdelhai et al., 2019). Our current result showed that ATF treatment suppressed the increases of peel firmness loss, electrolyte leakage, O_2_^•–^ production rate, and MDA content in *P. italicum*-infected Ponkan fruit, indicating that ATF treatment controlled blue mold development in Ponkan fruit by reducing *P. italicum*-induced oxidative damage. Many similar studies indicated lower firmness loss, electrolyte leakage, and MDA accumulation following the exogenous treatment of linalool in *Botrytis cinerea-*infected “Akihime” strawberry (Xu et al., 2019b), benzothiadiazole (BTH) in *Botryosphaeria dothidea*-infected “Fuji” apple (Huang et al., 2021b), and L-lysine in *Alternaria alternata*-infected “Zaosu” pear (Liu et al., 2021), demonstrating that these PAAs treatments enhanced the maintenance of cell wall structure and cell membrane stability and triggered the resistance against fungal infection in fruits.

When infected by fungus, an excessive accumulation of ROS, especially O_2_^•–^, can give rise to oxidative damage and cell wall degradation. Ordinarily, with the gradual increase of O_2_^•–^ production, the polysaccharides from plant cell walls were consumed and degraded, leading to weakened or loss of plant defense barrier against postharvest fungus. A concomitant increase in of O_2_^•–^ production rate in the control of *P. italicum-*infected Ponkan fruit suggested the occurrence of oxidative damage arising from excessive ROS accumulation, and it was considerably delayed by ATF treatment (Figure 1), which is essential to reserve the defense barrier of Ponkan fruit. This result was closely consistent with the result reported by Xue et al. (2020), who investigated and found that exogenous acetylsalicylic acid (ASA) treatment reduced *Fusarium sulphureum*-induced oxidative damage by suppressing O_2_^•–^ production and inducing fusarium rot resistance in “Manao” muskmelon (*Cucumis melo* L.) fruit. Nevertheless, accumulating studies have well demonstrated that PAAs could increase the activity of the antioxidant system scavenging excessive ROS in fungus-infected fruits (Segal and Wilson, 2018; Duan et al., 2021); these ROS-scavenging mechanisms involve the increase of enzymatic antioxidant activities and the induction of non-enzymatic antioxidant amounts (Xue et al., 2020; Zhang et al., 2020; Chen et al., 2022). For this reason, the maintenance of high antioxidant status is conducive to delaying the progression of fungal infection in fruits. In this study, the activities of antioxidant enzymes (e.g., SOD, CAT, and POD) and the expression level of their corresponding genes in *P. italicum*-infected Ponkan fruit were enhanced by ATF treatment (Figure 4). As an essential enzyme of the enzymatic antioxidant system, SOD acts as the first step of the ROS-scavenging process to dismutate O_2_^•–^ into hydrogen peroxide (H_2_O_2_) and O_2_; subsequently, the dismutation-produced H_2_O_2_ was decomposed into H_2_O by the synergistic efforts from CAT, POD, and AsA-GSH cycle-related enzymes. The higher levels of SOD, CAT, and POD activities and their related gene expression contributed to the enhancement of host resistance and the indirect attenuation of oxidative damage, which reduced blue mold development and lowered O_2_^•–^ production in Ponkan fruit. Our results were fully similar to other reports, in which SOD, CAT, and POD activities were increased and their enhanced corresponding gene expression was beneficial to trigger host resistance of harvested fruits, as observed in the β-aminobutyric acid-treated “Washington” orange fruit infected with *P. digitatum* (Elsherbiny et al., 2021), carvacrol-treated pummelo fruit infected with *D. citri* (Chen et al., 2022), *Pichia anomala*-treated “Red Globe” table grape infected with *P. expansum* (Godana et al., 2021), and pectic oligosaccharides-treated “Rio Red” grapefruit response to chilling stress at 2°C (Vera-Guzmán et al., 2019), revealing a regulatory mechanism of ATF involving the development of postharvest blue mold by mitigating O_2_^•–^ production and preserving high-antioxidant enzyme (e.g., SOD, CAT, and POD) activities and related gene expression in *P. italicum*-infected Ponkan fruit during the postinoculation period.

Apart from SOD, CAT, and POD, AsA-GSH cycle plays its prominent role in eliminating H_2_O_2_ and reducing ROS stress. Both non-enzymatic antioxidants of AsA and GSH have their redox potentials to detoxify H_2_O_2_ and maintain ROS homeostasis, and the maintenance of their redox state is contributed to enhancing host resistance of plants response to fungal infection, which directly depends on the activities of six important AsA-GSH cycle-related enzymes, namely, APX, GR, MDHAR, DHAR, GST, and GXP that participate in eliminating harmful H_2_O_2_ (Hasanuzzaman et al., 2019; Sadeghi et al., 2020; Zhu et al., 2022). In this cycle, as an electron donor, AsA helps APX to convert excessive H_2_O_2_ into H_2_O and generate monodehydroascorbate (MDHA) that regenerates AsA and is spontaneously converted into DHA with the aid of MDHAR; due to the participation of GSH, DHAR catalyzed the conversion of the produced DHA to GSSG and generated AsA; the conversion of GSH to GSSG needed the catalysis of GPX and GST, which is again restored to GSH with the help of GR (Hasanuzzaman et al., 2019; Xue et al., 2020). Higher levels of AsA and GSH content, as well as GSH/GSSG ratio, reflect the redox state of plant cells, and higher antioxidant capacity is beneficial for the maintenance of normal cell functions and the improvement of plant host resistance to fungal infection and abiotic stress (Sadeghi et al., 2020; Wang et al., 2020). In this study, in comparison to the control fruit, ATF-treated Ponkan fruit exhibited high levels of AsA and GSH content (Figures 2A,D), along with lower DHA and GSSG content (Figures 2B,E), enhanced the rise of AsA/DHA ratio (Figure 2C) and delayed the decline of GSH/GSSG ratio (Figure 2F), as well as induced the increase of antioxidant capacity (Figure 3); the activities of APX, GR, DHAR, GPX, and GST in *P. italicum*-infected Ponkan fruit were all enhanced by ATF treatment (Figures 5A,C,G,I,K), which contributed to the balance of AsA-GSH cycle and the maintenance of antioxidant capacity in postharvest Ponkan fruit’s response to *P. italicum* infection. Meanwhile, higher redox states in *P. italicum-*infected Ponkan fruit treated with ATF may arise from the upregulated expression of six AsA-GSH cycle-related genes (*APX6*, *GR2*, *MDHAR5*, *DHAR2*, *GPX2*, and *GST3*). There is accumulating evidence that exogenous PAAs application triggered the resistance in horticultural fruits by keeping a higher redox state through increasing the enzyme activities and gene expressions in AsA-GSH cycle during the postharvest period. For example, Wang et al. (2020) reported that the induced priming resistance against *B. cinerea* in “Kyoho” grapes by exogenous β-aminobutyric acid (BABA) treatment, ascribed to increase the levels of AsA and GSH or antioxidant capacity, as evidenced by upregulated expression levels of *VvGXP*, *VvGR*, *VvMDHAR*, *VvDHAR*, and *VvAPX*, resulted in maintaining intercellular redox status, revealed by higher NADPH content. Also, Chen et al. (2022) pointed out that the *Phomopsis* stem-end rot development in the harvested pummelo fruit, resulting from lower ROS-scavenging ability and more awful antioxidant system, coincided with higher O_2_^•–^ production and H_2_O_2_ accumulation arose from lower non-enzymatic antioxidant amounts accompanied by lower activities of APX, GR, SOD, CAT, and POD, leading to lower cell membrane integrity, evidenced by the tremendous increases in electrolyte leakage and MDA accumulation. Therefore, it can be inferred that ATF has great potential in alleviating *P. italicum*-induced oxidative damage in harvested Ponkan fruit by driving the AsA-GSH cycle.

## Conclusion

**FIGURE 6:**
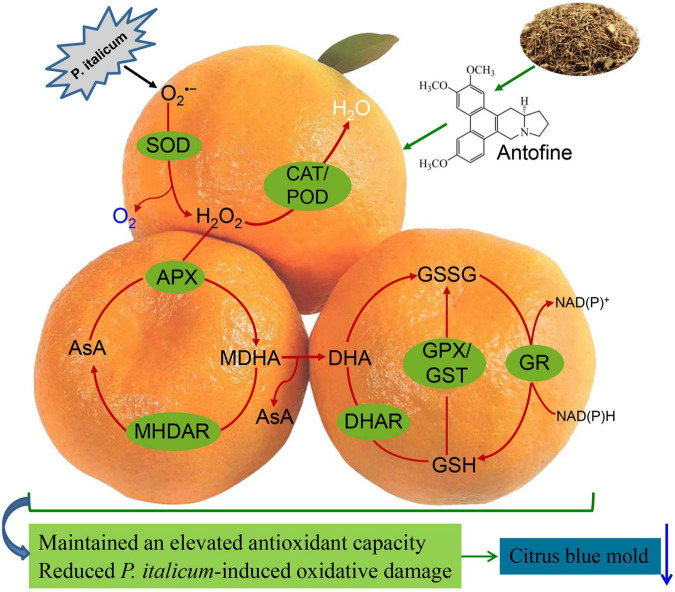
The possible mechanism of ATF treatment triggered the resistance and maintained a higher redox state by driving AsA-GSH cycle and ROS-scavenging system in *P. italicum*-infected Ponkan fruit.

In summary, ATF treatment could effectively control blue mold in harvested Ponkan fruit through the activation of the ROS-scavenging system. Moreover, the increases of electrolyte leakage, O_2_^•–^ production, MDA accumulation, and lipid membrane oxidation during *P. italicum* infection were significantly delayed by ATF treatment. The effect of ATF on the triggered resistance in harvested Ponkan fruit is linked to the alleviation of *P. italicum-*induced O_2_^•–^ overproduction by activating the ROS-scavenging mechanisms, evidenced by the induction of non-enzymatic antioxidant amounts and the increase of enzymatic antioxidant activities. The effects of the oxidative damage reduced the progression of *P. italicum* infection, as shown by the induction of the increase in GSH content, along with lower DHA and GSSG content, and delayed the declines in AsA content as well as GSH/GSSG ratio, and enhanced levels of ROS-scavenging enzyme activities and gene expression in the ATF-treated Ponkan fruit (Figure 6). Collectively, it can be concluded that ATF treatment might have a great potential in gaining the triggered resistance in citrus fruits during postharvest storage.

## Data Availability Statement

The raw data supporting the conclusions of this article will be made available by the authors, without undue reservation.

## Author Contributions

XP performed the experiments and wrote the original draft of this manuscript with the assistance of YZ, CW, and ZG. CW carried out data curation and participated in the review of this manuscript. CC designed the research and provided financial support. JC provided an experimental platform. All authors have read and approved the submitted version of the article.

## Conflict of Interest

The authors declare that the research was conducted in the absence of any commercial or financial relationships that could be construed as a potential conflict of interest.

## Publisher’s Note

All claims expressed in this article are solely those of the authors and do not necessarily represent those of their affiliated organizations, or those of the publisher, the editors and the reviewers. Any product that may be evaluated in this article, or claim that may be made by its manufacturer, is not guaranteed or endorsed by the publisher.

## Supplementary Material

Supplementary Figure 1 The inhibitory effect of ATF (0 ×, 1.25 ×, 2.5 ×, 5 ×, and 10 × MFC) treatment on blue mold development in harvested Ponkan fruit. Blue mold development **(A)**, lesion diameter **(B)**, and control efficiency **(C)** were measured after 6 days of *P. italicum* incubation at 27°C. Bars indicate the mean of 12 fruits ± SD and means labeled with different letters were significantly different at *P* < 0.01 according to the Duncan’s multiple range test.
