# Supplementary figures and images for: Antofine Triggers the Resistance Against Penicillium italicum in Ponkan Fruit by Driving AsA-GSH Cycle and ROS-Scavenging System
Source: Front Microbiol. 2022 Apr 12;13:874430. doi: 10.3389/fmicb.2022.874430 (PMC9039625; doi:10.3389/fmicb.2022.874430)

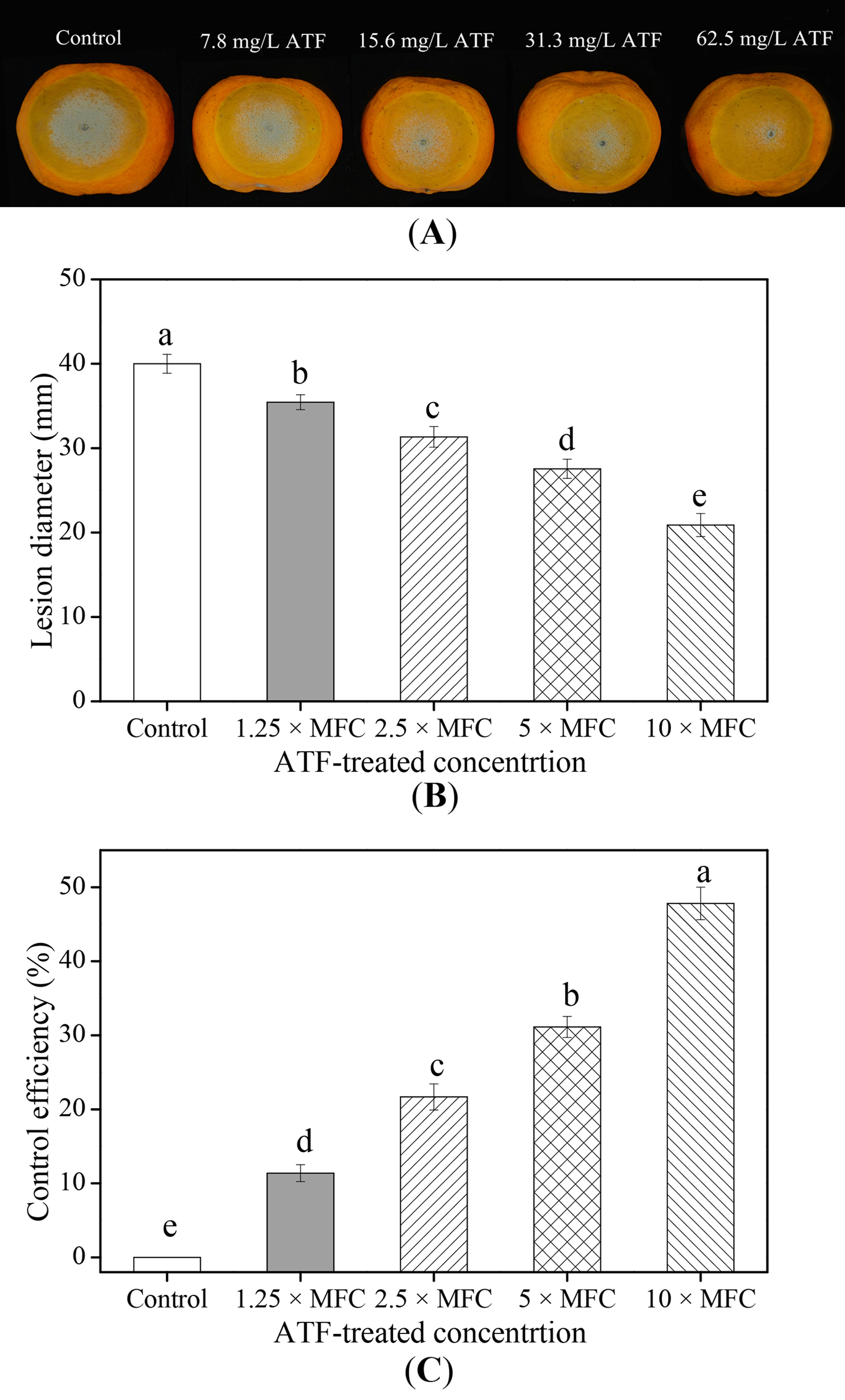

Supplement: Supplementary Figure 1 — The inhibitory effect of ATF (0 ×, 1.25 ×, 2.5 ×, 5 ×, and 10 × MFC) treatment on blue mold development in harvested Ponkan fruit. Blue mold development (A), lesion diameter (B), and control efficiency (C) were measured after 6 days of P. italicum incubation at 27°C. Bars indicate the mean of 12 fruits ± SD and means labeled with different letters were significantly different at P < 0.01 according to the Duncan’s multiple range test. [file Image_1.JPEG]
