# Supplementary material for: Antofine Triggers the Resistance Against Penicillium italicum in Ponkan Fruit by Driving AsA-GSH Cycle and ROS-Scavenging System
Source: Front Microbiol. 2022 Apr 12;13:874430. doi: 10.3389/fmicb.2022.874430 (PMC9039625; doi:10.3389/fmicb.2022.874430)
Supplement: Supplementary file 2 [file Table_1.DOC]

**Supplementary Table 1** Primer sequences used in the qRT-PCR analysis.

| Gene | Primer sequence (5’-3’) | Gene ID | Ponkan v1.0 |
| --- | --- | --- | --- |
| *MnSOD2* | forward: ACTACAACAAGGCGGTCGAGC | LOC102616226 | Ponkan7g_002820 |
|  | reverse: TGAGTGGTTGACATGACCTCCG |  |  |
| *CAT1* | forward: AGCCAGTTGGACGCTTGGT | LOC102615999 | Ponkan2g_014350 |
|  | reverse: TGGGGGCATTAACTGGAAGCA |  |  |
| *POD4* | forward: AGGCGTTGTTTCCTGTGCTGAT | LOC102629997 | Ponkan1g_008060 |
|  | reverse: CCTGTTTGCTGTTCTGCTGTCT |  |  |
| *APX6* | forward: CGA AATGTGCGGCGTCGG | LOC18042802 | Ponkan2g_006070 |
|  | reverse: CCCTTCGAGGCCACTCCTC |  |  |
| *GR2* | forward: ACTAATAAAGGAACAGTTGATGGCTTCTC | LOC102611650 | Ponkan5g_040100 |
|  | reverse: TCAACCTCTATAGCTCCATTCTTGGTC |  |  |
| *MDHAR5* | forward: TCGAGAATTTGTGATCGTTGGT | LOC102615220 | Ponkan5g_022520 |
|  | reverse: TCCATCAGCCATTCCATGTTC |  |  |
| *DHAR2* | forward: CAAGCCTCAATGGTTTATGGAGAT | LOC102620936 | Ponkan7g_004490 |
|  | reverse: CAGAGTCAGCCACCCACTTGT |  |  |
| *GPX2* | forward: CAGTGTGGCTTGACCAATTCAA | LOC102616580 | Ponkan5g_023250 |
|  | reverse: CCCCTCCTTATCCACCAAGAAC |  |  |
| *GST3* | forward: TGGTCGGGGAAAGGAGAAGA | LOC102621779 | Ponkan5g_037340 |
|  | reverse: GTGAATGGAACCAACGCCAC |  |  |
| *β-Actin* | forward: GCTCCAAGCAGCATGAAGATCAAGG | LOC102577980 | Ponkan1g_003790 |
|  | reverse: TGCTGGAAGGTGCTGAGGGA |  |  |
